# Thermal conductivity and viscosity of self-assembled alcohol/polyalphaolefin nanoemulsion fluids

**DOI:** 10.1186/1556-276X-6-274

**Published:** 2011-03-31

**Authors:** Jiajun Xu, Bao Yang, Boualem Hammouda

**Affiliations:** 1 Department of Mechanical Engineering, University of Maryland, College Park, MD 20742, USA; 2 National Institute of Standards and Technology, Center for Neutron Research, Gaithersburg, MD 20899, USA

## Abstract

Very large thermal conductivity enhancement had been reported earlier in colloidal suspensions of solid nanoparticles (i.e., nanofluids) and more recently also in oil-in-water emulsions. In this study, nanoemulsions of alcohol and polyalphaolefin (PAO) are spontaneously generated by self-assembly, and their thermal conductivity and viscosity are investigated experimentally. Alcohol and PAO have similar thermal conductivity values, so that the abnormal effects, such as particle Brownian motion, on thermal transport could be deducted in these alcohol/PAO nanoemulsion fluids. Small angle neutron-scattering measurement shows that the alcohol droplets are spheres of 0.8-nm radius in these nanoemulsion fluids. Both thermal conductivity and dynamic viscosity of the fluids are found to increase with alcohol droplet loading, as expected from classical theories. However, the measured conductivity increase is very moderate, e.g., a 2.3% increase for 9 vol%, in these fluids. This suggests that no anomalous enhancement of thermal conductivity is observed in the alcohol/PAO nanoemulsion fluids tested in this study.

## Introduction

Nanofluids, i.e., colloidal suspensions of solid nanoparticles, and more recently, nanoemulsion fluids have attracted much attention because of their potential to surpass the performance of conventional heat transfer fluids [1-22]. The coolants, lubricants, oils, and other heat transfer fluids used in today's thermal systems typically have inherently poor heat transfer properties which have come to be reckoned as the most limiting technical challenges faced by a multitude of diverse industry and military groups. A number of studies have been conducted to investigate thermal properties of nanofluids with various nanoparticles and base fluids. However, the scientific community has not yet come to an agreement on the fundamental effects of nanoparticles on thermal conductivity of the base fluids. For example, many groups have reported strong thermal conductivity enhancement beyond that predicted by Maxwell's model in nanofluids [1,2,23,24]. Consequently, several hypotheses were proposed to explain those unexpected experimental results, including particle Brownian motion, particle clustering, ordered liquid layer, and dual-phase lagging [18,21,25-28]. Recently, however, an International Nanofluid Property Benchmark Exercise reported that no such anomalous enhancement was observed in nanofluids [22].

In this study, nanoemulsion fluids of alcohol in polyalphaolefin (PAO) are employed to investigate the effects of nanodroplets on the fluid thermal conductivity and viscosity. These fluids are spontaneously generated by self-assembly. The dependence of thermal conductivity and viscosity on droplet concentration has been obtained experimentally in these nanoemulsion fluids. The droplet size is determined by the small angle neutron-scattering (SANS) technique.

### Nanoemulsion heat transfer fluids

Nanoemulsion fluids are suspensions of liquid nanodroplets in fluids, which are part of a broad class of multiphase colloidal dispersions [17,29,30]. The droplets typically have length scale <100 nm. The nanoemulsion fluid can be formed spontaneously by self-assembly without need of external shear-induced rupturing. These nanodroplets are in fact swollen micelles in which the outer layer is composed of surfactant molecules having hydrophilic heads and hydrophobic tails. It should be stressed that the nanoemulsion fluids are thermodynamically stable, unlike conventional (macro) emulsions.

Nanoemulsion fluids could serve as a model system to investigate the effects of particles on thermophysical properties in nanofluids because of their inherent features: (1) their superior stability, (2) their adjustable droplet size, (3) thermal conductivity and volume concentration of droplets can be accurately determined, etc.

**Figure 1:**
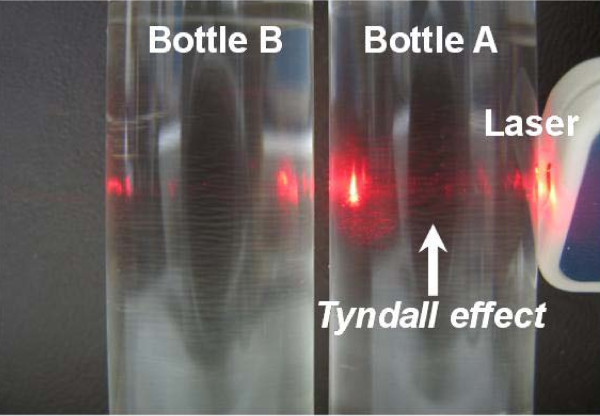
**Alcohol/PAO nanoemulsion fluids (Bottle A) and pure PAO (Bottle B)**. Liquids in both bottles are transparent. The Tyndall effect (i.e., a light beam can be seen when viewed from the side) can be observed only in Bottle A when a laser beam is passed through Bottles A and B. Pictures taken using a Canon PowerShot digital camera.

In this study, nanoemulsions of alcohol in PAO are formed, in which the alcohol droplets (Sigma-Aldrich Co., MO , USA) are stabilized by the surfactant molecules sodium *bis*(2-ethylhexyl) sullfosuccinate (Sigma Aldrich) that have hydrophilic heads facing inward and hydrophobic tails facing outward into the base fluid PAO (Chevron Phillips Chemical Company LP, TX, USA). Figure 1 shows the picture of the prepared alcohol/PAO nanoemulsion fluids and the pure PAO. The alcohol/PAO nanoemulsion fluid is optically transparent, but scatters light due to the Tyndall effect. PAO is widely used as heat transfer fluid and lubricant, and is able to remain oily in a wide temperature range due to the flexible alkyl-branching groups on the C-C backbone chain. Alcohol is chosen as the dispersed phase because it has a thermal conductivity close to that of PAO, *k*_PAO _= 0.143 W/mK and *k*_alcohol _= 0.171 W/mK, at room temperature [31,32], so that the conductivity increase predicted from the effective medium theory would be minimized in such nanoemulsion fluids, and the contribution from other sources such as particle Brownian motion and dual-phase lagging could be deducted.

## Results and discussion

### SANS measurement

**Figure 2:**
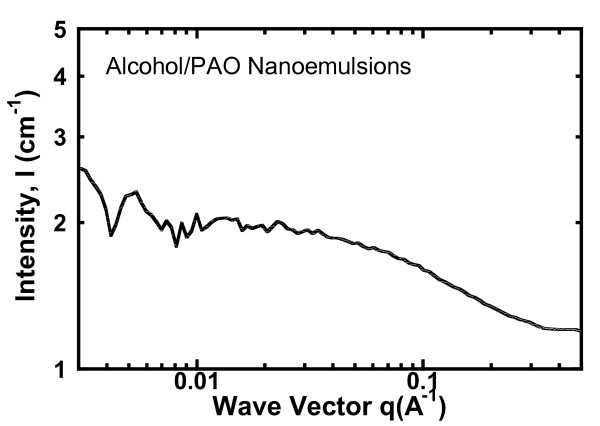
**SANS curve (scattering intensity *I *versus scattering vector *q*) for the alcohol/PAO nanoemulsion fluids with 9 vol%**. SANS measurement was made on the NG-3 beamline at NIST.

SANS measurements are carried out for the *in situ *determination of the size of droplets in the nanoemulsion fluids. Unlike the conventional dynamic light scattering, the SANS can be applied to the "concentrated" colloidal suspensions (e.g., >1 vol%) [33,34]. In our SANS experiment, samples are prepared using deuterated alcohol to achieve the needed contrast between the droplets and the solvent. SANS measurements are conducted on the NG-3 (30 m) beamline at the NIST Center for Neutron Research (NCNR) in Gaithersburg, MD. Samples are loaded into2-mm quartz cells. Figure 2 shows the SANS data, the scattering intensity *I *versus the scattering vector *q *= 4π sin(θ/2)/λ, where λ is the wavelength of the incident neutrons, and θ is the scattering angle. The approximation *q *= 2πθ/λ is used for SANS (due to the small angle θ). The analysis of the SANS data suggests that the inner cores of the swollen micelles, i.e., the alcohol droplets, are spherical and have a radius of about 0.8 nm for 9 vol%. The error in droplet size is about 10%. The SANS data were processed using the IGOR software under the protocol from NCNR NIST.

### Thermal conductivity characterization

A technique, named the 3ω-wire method, has been developed to measure the thermal conductivity of liquids [12,35]. Most of published thermal conductivity data on the nanofluids were obtained using the hot-wire method, which measures the temperature response of the metal wire in the time domain [36]. Our 3ω-wire method is actually a combination of the 3ω-wire and the hot-wire methods. Similar to the hot-wire method, a metal wire suspended in a liquid acts both as a heater and a thermometer. However, the 3ω-wire method determines the fluid conductivity by detecting the dependence of temperature oscillation on frequency, instead of time. In the measurement, a sinusoidal current at frequency ω is passed through the metal wire and then a heat wave at frequency 2ω is generated in the liquid. The 2ω temperature rise of the wire can be deduced by the voltage component at frequency 3ω. The thermal conductivity of the liquid, *k*, is determined by the slope of the 2ω temperature rise of the metal wire [12,37]:(1)

where *p *is the applied electric power, *ω *is the frequency of the applied electric current, *l *is the length of the metal wire, and *T*_2ω _is the amplitude of temperature oscillation at frequency 2ω in the metal wire. One advantage of this 3ω-wire method is that the temperature oscillation can be kept small enough (below 1 K, compared to about 5 K for the hot-wire method) within the test liquid to retain constant liquid properties. Calibration experiments were performed for hydrocarbon (oil), fluorocarbon, and water at atmospheric pressure. The literature values were reproduced with an error of <1%.

**Figure 3:**
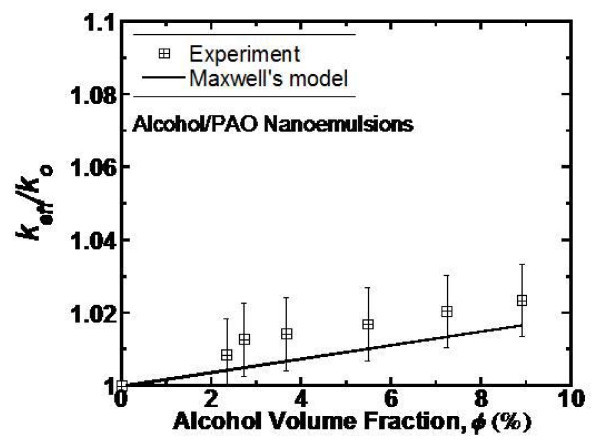
**Relative thermal conductivity of the alcohol/PAO nanoemulsion fluids versus alcohol volumetric fraction**. The prediction by the Maxwell equation is shown for comparison.

Figure 3 shows the relative thermal conductivity as a function of the loading of alcohol nanodroplets in alcohol/PAO nanoemulsion fluids at room temperature. The prediction by the Maxwell model is also plotted in Figure 3 for comparison. The relative thermal conductivity is defined as *k*_eff_*/k*_o_, where *k*_o _and *k*_eff _are the thermal conductivities of the base and nanoemulsion fluids, respectively. The PAO thermal conductivity is experimentally found to be 0.143 W/m K at room temperature, which compares well with the literature value [32]. It can be seen in this figure that the relative thermal conductivity of the alcohol/PAO nanoemulsion fluids appears to be linear with the loading of alcohol nanodroplets over the range from 0 to 9 vol%. However, the magnitude of the conductivity increase is rather moderate in the fluids, e.g., a 2.3% increase for 9 vol% loading.

The effective medium theory reduces to Maxwell's equation for suspensions of well-dispersed, non-interacting spherical particles [22,38]:(2)

where *k*_o _is the thermal conductivity of the base fluid, *k*_p _is the thermal conductivity of the particles, and ϕ is the particle volumetric fraction. Equation (2) predicts that the thermal conductivity enhancement increases approximately linearly with the particle volumetric fraction for dilute nanofluids or nanoemulsion fluids (e.g., ϕ <10%), if *k*_p _>*k*_o _and the particle shape remains unchanged. The solid line in Figure 3 represents the relative thermal conductivity evaluated from Equation (2). It can be seen that the measured thermal conductivity is in good agreement with the prediction of Maxwell's equation in the alcohol/PAO nanoemulsion fluids. The very small increase in thermal conductivity (<2.3%) is due to the fact that the thermal conductivity of alcohol is very slightly larger than that of PAO, *k*_PAO _= 0.143 W/mK, and *k*_alcohol _= 0.171 W/mK at room temperature. No strong effects of Brownian motion on thermal transport are found experimentally in those fluids although the nanodroplets are extremely small, around 0.8 nm.

### Viscosity characterization

Unlike the thermal conductivity, the viscosity of the alcohol/PAO nanoemulsion fluids is found to be altered significantly because of the dispersed alcohol droplets. A commercial viscometer (Brookfield DV-I Prime) is used for the viscosity measurement. The dynamic viscosity is found to be 7.3 cP in the pure PAO, which compares well with the literature value [32].

**Figure 4:**
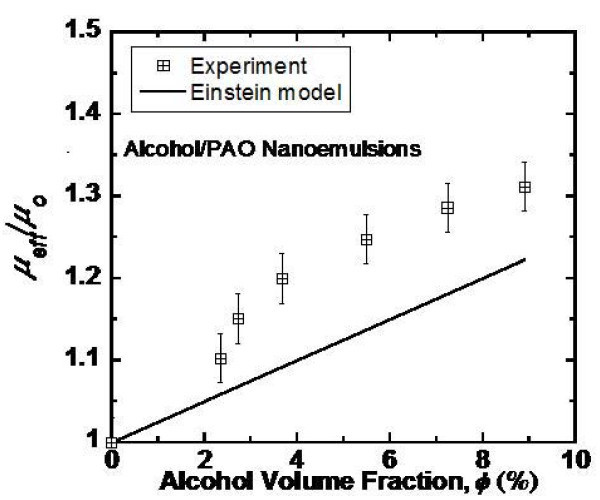
**Relative dynamic viscosity of the alcohol/PAO nanoemulsion fluids versus alcohol volumetric fraction**. The prediction by the Einstein equation is shown for comparison.

Figure 4 shows the relative dynamic viscosity, μ_eff_/μ_o_, for the alcohol/PAO nanoemulsion fluids with varying alcohol loading. An approximately linear relationship is observed between the viscosity increase and the loading of alcohol nanodroplets in the range of 0-9 vol%, a trend similar to thermal conductivity plotted in Figure 3. However, the relative viscosity is found to be much larger than the relative conductivity if compared at the same alcohol loading. For example, the measured viscosity increase is 31% for 9 vol% alcohol loading, compared to a 2.3% increase in thermal conductivity. It is worth noting that the viscosities of the pure PAO and the alcohol/PAO nanoemulsion fluids have been measured at spindle rotational speed ranging from 6 to 30 rpm and exhibits a shear-independent characteristic of Newtonian fluids.

The viscosity increase of dilute colloids can be predicted using the Einstein equation, μ_eff_/μ_0 _= 1 + 2.5φ [39]. This equation, however, underpredicts slightly the viscosity increase in the alcohol/PAO nanoemulsion fluids, as can be seen in Figure 4. This discrepancy is probably because the droplet volume fraction, ϕ, used in the viscosity calculation does not take into account the surfactant layer outside the alcohol core. That is, the actual volume fraction of droplets should be larger than the fraction of alcohol in the alcohol/PAO nanoemulsion fluids.

## Conclusion

The nanoemulsion fluids of alcohol in PAO are employed to investigate the effects of the dispersed droplets on thermal conductivity and viscosity. Alcohol and PAO have similar thermal conductivity values at room temperature and are physically immiscible. SANS measurements are conducted for the *in situ *determination of the droplet size in the nanoemulsion fluids. The fluid thermal conductivity is measured using the 3ω-wire method. As predicted by the classical Maxwell model, the increase in thermal conductivity is found to be very moderate, about 2.3% for 9 vol% loading, in the alcohol/PAO nanoemulsion fluids. This suggests that the thermal conductivity enhancement due to particle Brownian motion is not observed experimentally in these nanoemulsion fluids although the nanodroplets are extremely small, around 0.8 nm in radius. Unlike thermal conductivity, the viscosities of the alcohol/PAO nanoemulsion fluids are found to increase significantly due to the dispersed alcohol droplets.

## Abbreviations

NCNR: NIST Center for Neutron Research; PAO: polyalphaolefin; SANS: small angle neutron scattering.

## Competing interests

The authors declare that they have no competing interests.

## Authors' contributions

JX did the synthetic and characteristic job, and participated in drafting the manuscript. BY conceived of the study, provided instruction on the experiment, and drafted the manuscript. BH performed the SANS measurement and assisted in data processing and analysis.

All authors read and approved the final manuscript
